# Temporary Unilateral Hearing Loss Impairs Spatial Auditory Information Processing in Neurons in the Central Auditory System

**DOI:** 10.3389/fnins.2021.721922

**Published:** 2021-11-01

**Authors:** Jennifer L. Thornton, Kelsey L. Anbuhl, Daniel J. Tollin

**Affiliations:** ^1^Department of Physiology and Biophysics, University of Colorado School of Medicine, Aurora, CO, United States; ^2^Center for Neural Science, New York University, New York, NY, United States

**Keywords:** sound localization, inferior colliculus, conductive hearing loss, interaural level difference, mutual information

## Abstract

Temporary conductive hearing loss (CHL) can lead to hearing impairments that persist beyond resolution of the CHL. In particular, unilateral CHL leads to deficits in auditory skills that rely on binaural input (e.g., spatial hearing). Here, we asked whether single neurons in the auditory midbrain, which integrate acoustic inputs from the two ears, are altered by a temporary CHL. We introduced 6 weeks of unilateral CHL to young adult chinchillas via foam earplug. Following CHL removal and restoration of peripheral input, single-unit recordings from inferior colliculus (ICC) neurons revealed the CHL decreased the efficacy of inhibitory input to the ICC contralateral to the earplug and increased inhibitory input ipsilateral to the earplug, effectively creating a higher proportion of monaural responsive neurons than binaural. Moreover, this resulted in a ∼10 dB shift in the coding of a binaural sound location cue (interaural-level difference, ILD) in ICC neurons relative to controls. The direction of the shift was consistent with a compensation of the altered ILDs due to the CHL. ICC neuron responses carried ∼37% less information about ILDs after CHL than control neurons. Cochlear peripheral-evoked responses confirmed that the CHL did not induce damage to the auditory periphery. We find that a temporary CHL altered auditory midbrain neurons by shifting binaural responses to ILD acoustic cues, suggesting a compensatory form of plasticity occurring by at least the level of the auditory midbrain, the ICC.

## Introduction

Conductive hearing loss (CHL) during development can change auditory system structure and function (see reviews by Moore and King, 2004; Tollin, 2010; Whitton and Polley, 2011). Early life exposure to CHL, particularly unilateral, can lead to impairments in binaural hearing, including sound localization accuracy and acuity as well as spatial unmasking, even after resolution of the CHL and hearing sensitivity in both ears returns to normal (Moore et al., 1991; Ludwig et al., 2019). Binaural hearing deficits can recover, but can take months or years. During recovery, a child may present as audiologically normal, yet have lingering difficulty with speech perception in noisy, reverberant environments. As language is often learned in such environments, these impairments may contribute to deficits in language acquisition which is often observed in children with a history of chronic CHL (for review, see Whitton and Polley, 2011). CHL induced in adulthood can also be detrimental to binaural hearing. For example, adults who experience prolonged periods of mild-moderate HL display worse performance on a binaural hearing task even long after corrective surgery (Hall and Derlacki, 1986; Ferguson et al., 1998). Decades of studies of the neural, anatomical and behavioral consequences of experimentally induced CHL in animal models have revealed effects related to the timing of onset, the duration, and the severity of the deprivation (Clopton and Silverman, 1977; Silverman and Clopton, 1977; Popescu and Polley, 2010; Keating and King, 2013; Polley et al., 2013; Keating et al., 2015; see Kumpik and King, 2019 for review). Regarding neural processing, these studies have generally examined how CHL alters basic neural response properties. However, it remains unclear how these alterations contribute to the persistent binaural hearing deficits. Here, we utilized the novel framework of information theory (Dayan and Abbott, 2001) to quantify how much information auditory midbrain neurons carry about a binaural cue to sound location (i.e., interaural level differences, ILD). We then examine how a temporary CHL induced in young adult animals alters neural information processing of binaural information, providing insight to underlying mechanisms of binaural function.

## Materials and Methods

All surgical and experimental procedures complied with the guidelines of the University of Colorado Anschutz Medical Campus Animal Care and Use Committees and the National Institutes of Health. Eleven young adult (∼P70; all female) chinchillas were used for the deprivation experiments while 19 normal-hearing animals (∼P70; 10 female, 9 male) were used for control data. This age was chosen as the head, pinnae, ear canal, and associated acoustical cues to sound location reach adult-like ranges at this time (Jones et al., 2011b). Similar to that described in Lupo et al. (2011), a small foam earplug (AO Safety, Indianapolis, IN, United States) was custom fit to the external ear canal of the animal and was then inserted into the left ear canal for 6 weeks. Foam earplugs (Lupo et al., 2011) produce similar spectrotemporal alterations of sound input to the cochlea as fluid in the middle ear (Thornton et al., 2012, 2013). Earplugs were checked daily to ensure stable placement within the ear canal.

### Cochlear Microphonic Recordings

Animals (CHL: *n* = 11; control: *n* = 19) were anesthetized and prepared for electrophysiology as described in Jones et al. (2011a). Animals were anesthetized with an intramuscular (i.m.) injection of ketamine hydrochloride (KetaVed, 30 mg/kg i.m.) and xylazine hydrochloride (TranquiVed, 5 mg/kg im); supplementary injections were administered to maintain an adequate level of anesthesia. Next, a hole (2–3 mm diameter) was made in each bulla through which a silver ball electrode was placed on the round windows and fixed in place with dental acrylic, resealing the bullae. The cochlear microphonic (CM) was differentially amplified, filtered, and visually verified by oscilloscope. To quantify the magnitude of the CHL due to the earplug, free-field CM measurements were taken for both the left (earplugged in CHL group) and right (no earplug) ears for three different conditions: with the earplug in place (left ear), after the earplug was removed, and the right ear (within-animal control). Stimuli consisted of 10–ms sinusoids (2.5–ms rise/fall, 5–ms plateau) with octave steps from 0.25 to 20 kHz. Each stimulus was presented at least 25 times with a 40–ms interstimulus period.

### Electrophysiological Recordings

Single unit, extracellular responses were recorded from neurons in the ICC as described in Jones et al. (2015). Briefly, extracellular electrical activity was measured with Parylene-coated tungsten microelectrodes (1–2 MΩ; Microprobe, Clarksburg, MD). Electrical activity was amplified (ISO-80, WPI, Sarasota, FL; Stanford Research Systems SRS 560, Sunnyvale, CA) and filtered (300–3,000 Hz). The colliculus was located stereotaxically and confirmation of ICC was determined by systematic changes in the characteristic frequencies (CFs) of neurons as the electrode was advanced. Candidate extracellular responses were isolated with a BAK amplitude-time window discriminator (model DDIS-1, Mount Airy, MD), and spike times were stored at a precision of 1μs via a TDT RV8. Neurons were studied if their single-unit spike waveforms exhibited good signal-to-noise ratio along with amplitude and temporal action potential morphology that was consistent from spike to spike. All recordings in the group of animals with CHL were performed the same day as the earplug removal.

Frequency-intensity response areas were measured with tone pips to estimate the CF and threshold. Next, neurons were classified in terms of their binaural input patterns in response to high level (20–30 dB re: threshold) CF tones. Briefly, neurons are given a classification that corresponds with the amount of excitation (or lack thereof, or “O”) that they receive from each ear. For example, a neuron that receives excitatory inputs from both the contralateral and ipsilateral ears is classified as EE (one excitatory input, “E,” from the contralateral ear and one excitatory input, “E,” from the ipsilateral ear). A neuron with excitation from the contralateral and inhibition from the ipsilateral ear is designated EI. And some neurons only responded to stimulation of the contralateral ear and are designated EO.

**FIGURE 1:**
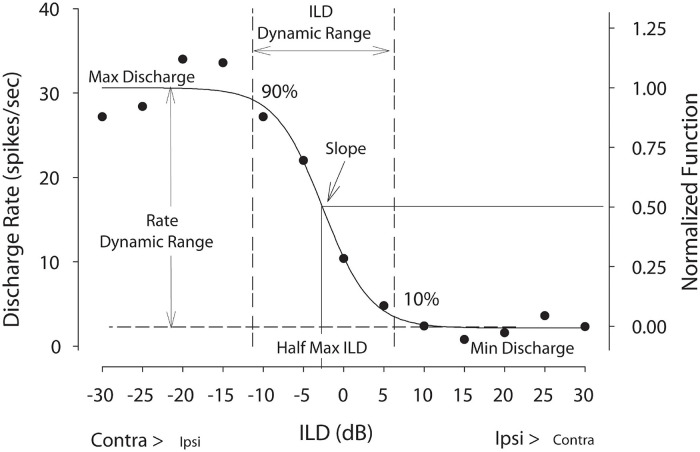
Example discharge rate vs. ILD neural tuning function. Raw spike rate data were fitted with a 4-parameter logistic function from which key tuning parameters were computed including half-maximal ILD—the ILD producing 50% of maximal firing—and ILD dynamic range—the range of ILDs producing 10–90% of maximal firing. Rate dynamic range is defined as the difference between the maximum and minimum rates from the fitted curves divided by the maximum rate. For population level analyses, firing rates were normalized. This figure was adapted from Jones et al. (2015).

Finally, in a smaller subset of neurons, ILD sensitivity was examined using 50 repetitions of 50-ms duration CF tones by holding the signal level to the contralateral ear (∼20 dB re: each neuron threshold) constant and varying the level at the ipsilateral ear from ≥ 25 dB below to 25 dB above ipsilateral threshold (5-dB steps). Following Brown and Tollin (2016), the rate vs. ILD for each neuron was fitted with a 4-parameter sigmoid, where rate (ILD) = y0 + α/(1-ex*p*(-(ILD-ILD0)/β)). Before fitting, the data were normalized to the maximum rate (Figure 1). The fits described the data for all neurons (*R* > 0.9); therefore, the fit parameters were used for all subsequent analysis. The parameters examined were half-max ILD (i.e., ILD at 50% of the maximal rate), rate-ILD slope (i.e., spikes/s/dB, not normalized; computed at half-max ILD), ILD dynamic range defined between 90 and 10% of maximum rate, and spike rate (or spike count) modulation defined as the difference between the maximum and minimum rate (or count) from the fitted rate-ILD function divided by the maximum rate (or count).

### Neural Information Analysis—Mutual Information Computation

The mutual information (MI) is a measure of the strength of the association between two random variables, such as a spike count, *r*, and a given stimulus, *S* (Dayan and Abbott, 2001). MI is given by


$$MI\left(r,S\right)=\sum_{i}\sum_{j}p\left(S_{j}\right)p\left\langle r_{i}|S_{j}\right\rangle log_{2}\left[\frac{p\left\langle r_{i}|S_{j}\right\rangle }{p\,\left(r_{i}\right)}\right]$$

where *p(Sj)* is the probability that the stimulus (S) had a particular value [S-values (i.e., ILDs) were presented with equal probability], *p(ri)* is the probability that the count was *r*_*i*_ at any value of *S*, and *p(ri| Sj)* is the probability that the count was *r*_*i*_ when the stimulus was *S*_*j*_. Intuitively, MI will be high when the count variability is larger when computed across different stimuli than the variability computed within single presentations of a particular stimulus. MI summarizes all information contained in a neuronal response into a single, meaningful number as measured in bits. The benefit of using MI as a measure of neural sensitivity is that it allows one to make very few assumptions about the form of the neural response properties. The MI represents the upper bound on the information that even the “best “decoder” could represent (Benichoux et al., 2017). Thus, if CHL changes the information carrying capacity of ICC neurons then the MI will capture and quantify it.

## Results

### Conductive Hearing Loss Due to Earplug Does Not Alter Auditory Periphery

To quantify the CHL caused by the earplug, as well as assay the function of the peripheral auditory system, sound-evoked CM responses were measured while the earplug was still in place and immediately after earplug removal (see Lupo et al., 2011; Thornton et al., 2012, 2013 for detailed methods). CM data from the right (unplugged) ear was used as a within-animal control, as unilateral CHL does not alter the CM in the non-occluded contralateral ear (Larsen and Liberman, 2010). The CHL was ∼10–15 dB for frequencies < 4 kHz increasing to ∼30 dB for frequencies > 4 kHz, consistent with Lupo et al. (2011). The magnitude of the CHL induced by earplugs is comparable to a CHL due to middle-ear effusion (Thornton et al., 2012, 2013). With earplugs, the mean CM thresholds across all frequencies and animals (n = 11) was 46.2 ± 7.1 dB. After removal of the earplug, CM thresholds were reduced to 30.7 ± 8.3 dB. Thus, the plug produced an across-frequency attenuation of 15.5 dB. A two-way repeated-measures ANOVA revealed that there was a significant decrease in attenuation after the earplug was removed [*F*_(__1, 10__)_ = 103.3, *p* < 0.0001]. There was no significant difference between the CM thresholds in the control ear and thresholds in the experimental ear after the earplug was removed [*F*_(__1, 15__)_ = 3.71, *p* = 0.073]. The return of CM thresholds to normal levels after earplug removal indicates that the hearing loss induced by the plug was reversible, from 0.25 to 20 kHz. These data indicate that the earplug-induced CHL produced a reversible hearing loss without damaging the auditory periphery.

### Unilateral Conductive Hearing Loss Does Not Alter Frequency Selectivity or Thresholds of Monaural and Binaural Sensitive Neurons

Monaural and binaural response properties were studied for a total of 323 IC neurons in normal control animals and 134 neurons from CHL animals after earplug removal. Neurons from the ICC contralateral to the previous CHL were analyzed.

The CFs for neurons with EE responses averaged 1.63 ± 2.0 kHz (median: 0.840, range: 0.140–8.95 kHz, *n* = 140) in controls and 1.76 ± 2.85 kHz (0.963, 0.320–13.77 kHz, n = 23) for neurons in the ICC contralateral to the CHL; CFs for EE neurons were not significantly different between control and experimental group [*t*_(__159)_ = –0.27, *p* = 0.79]. Thresholds for EE neurons averaged 14.44 dB SPL (median: 10, range: –15–50 dB, *n* = 140) for controls and 18.0 ± 12.5 dB SPL (15, 0–40 dB, *n* = 23) for neurons contralateral to the CHL; thresholds were not significantly different ([*t*_(__159)_ = –1.04, *p* = 0.3]. The CFs for neurons with EO responses averaged 5.1 ± 4.34 kHz (median: 4.2, range: 0.08–17.3 kHz, *n* = 95) in controls and 5.68 ± 3.42 kHz (5.15, 0.162–15.7 kHz, *n* = 82) for neurons contralateral to the CHL; CFs for EO neurons were not significantly different [*t*_(__175)_ = –0.97, *p* = 0.33]. Thresholds for EO neurons averaged 20.91 ± 14.18 dB SPL (median: 15, range: –10–55 dB SPL, *n* = 95) in controls and 23.84 ± 13.8 dB SPL (20, 0–60 dB SPL, *n* = 82) for neurons contralateral to the CHL; thresholds for EO neurons were not significantly different [*t*_(__175)_ = –1.38, *p* = 0.17]. Finally, CFs for neurons with EI responses averaged 7.25 ± 4.07 kHz (median: 6.28, range: 0.795–24.25 kHz, *n* = 90) in controls and 6.0 ± 3.56 kHz (5.38, 0.807–14.37 kHz, *n* = 26) for neurons contralateral to the CHL; CF for EI neurons were not significantly different [*t*_(__114)_ = 1.41, *p* = 0.16]. Thresholds for neurons with EI responses averaged 24.22 ± 12.36 dB SPL (median: 25, range: −10–55 dB SPL, *n* = 90) in controls and 29.62 ± 12.64 dB SPL (30, 10–50, *n* = 26) for neurons contralateral to the CHL; thresholds for EI neurons were not significantly different [*t*_(__114)_ = −1.95, *p* = 0.054].

### Distribution of Monaural and Binaural Neural Responses Is Altered by Unilateral Conductive Hearing Loss

While there were no significant differences with the range of CFs or thresholds of ICC neurons between groups, there were substantial changes in the distributions of monaural and binaural responses. In controls, 42.7% of the neurons were EE, 29.4% EO and 27.9% EI while in animals with unilateral CHL, the proportions were 17.6% for EE, 62.6% for EO and 19.8% for EI. A chi-square test indicated that there was a significant difference in the proportions of monaural and binaural categories due to the CHL [χ^2^(2, *N* = 457) = 72.13, *p* < 0.0001]. This suggests that the unilateral CHL shifted ICC neurons (contralateral to the previous CHL) from primarily binaurally responsive units (i.e., EE, EI) to primarily monaural responsive units (i.e., EO).

### Neural Coding of Interaural-Level-Difference Cues Is Altered by Unilateral Conductive Hearing Loss

**FIGURE 2:**
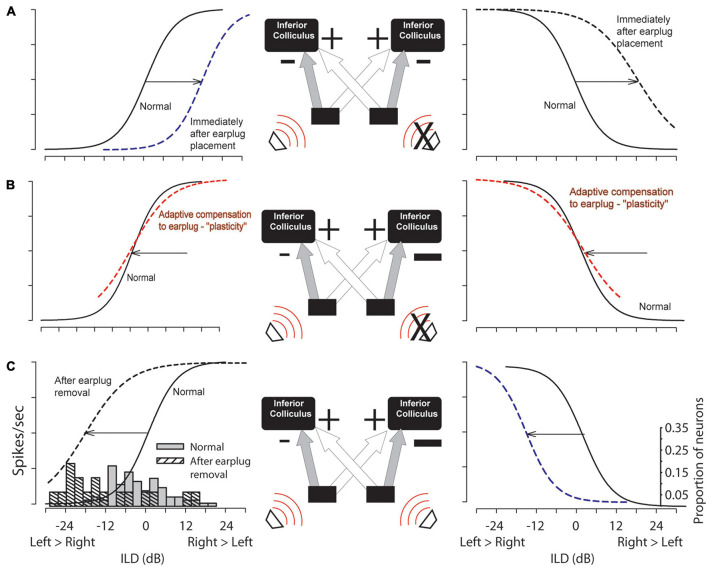
Hypothesized changes due to CHL (right ear, “X”) in circuit function and the sensitivity to ILDs in the left (contralateral to earplug) and right (ipsilateral to earplug) ICC are shown in **(A,B)** with empirical data shown in **(C)**. The simplified circuit shows ipsilateral inhibitory (“–”) and contralateral excitatory (“+”) inputs to the ICC (strengths indicated by sizes of the symbols). **(A)** Relative to ILD coding in normal hearing controls (solid lines) ILD coding shifts to the right due to earplug insertion (dashed). **(B)** Hypothetical shifts in ILD coding if circuit plasticity has compensated for unilateral earplug over time. **(C)** Upon earplug removal, ILD coding shifts left due to the CHL-induced altered circuitry. Histograms: empirical half-max ILDs form ICC neurons in normal hearing controls (*n* = 31) and experimental group after earplug removal (*n* = 19).

Figure 1 illustrates the parameters of rate-vs.-ILD functions considered in this section. Half-max ILD values were compared between normal-hearing control animals and animals that received a unilateral earplug as adults. A shift in half-max ILD indicates a shift in the entire rate-ILD curve, signifying that that specific neuron encodes a different range of ILDs. For control animals, the mean half-max ILD was 1.9 ± 8.3 dB (*n* = 31 units), with a median value of 0.94 dB and a range of from −20.6 to 17.1 dB (Figure 2C, left and right panels, gray bars). For CHL animals, neurons in the ICC contralateral to the CHL ear (Figure 2C, left panel) exhibited a shift in the mean half-max ILD value to −10.26 ± 12.2 dB (*n* = 19 neurons) with a median value of −14.1 dB. The overall range of ILDs encoded by these neurons was shifted toward negative ILDs, ranging from −28.4 to 15.0 dB (Figure 2C, left panel, gray hatched bars). Relative to controls, the CHL produced an effective shift in ILD coding of 12.2 dB for neurons contralateral to the CHL. An unpaired *t*-test indicated a significant difference in half-max ILDs between normal and earplugged neurons [*t*_(__48)_ = 3.85, *p* = 0.0004].

CHL also altered the ILD dynamic range of the rate-ILD curves. The mean ILD dynamic range for control neurons was 26.1 ± 10.1 dB (median: 25.7 dB). Following CHL, neurons in the ICC contralateral to the CHL displayed a mean dynamic range of 19.4 ± 9.8 dB (median: 17.6 dB), which was significantly lower than control neurons [*t*_(__48)_ = 2.24, *p* = 0.031].

The slopes of the ILD functions in the ICC contralateral to the hearing loss were not significantly different between normal animals that animals that had a unilateral earplug-induced CHL [*t*_(__48)_ = −1.75, *p* > 0.05]. Across ICC neurons in normal animals, the average rate-ILD slope was 2.34 ± 1.97 sp/s/dB with a median value of 2.06 sp/s/dB while in neurons contralateral to the CHL, the average rate-ILD slope was 2.9 ± 1.8 sp/s/dB with a median value of 2.4 sp/s/dB.

Finally, CHL altered the modulation of discharge rate due to ILD. In control neurons, the firing rate was modulated by 82 ± 16% (difference between unnormalized maximum and minimum rates derived from the fitted function divided by the maximum) with presentation of a range of ILD values (± 25 dB to the ipsilateral ear). Neurons contralateral to the CHL were modulated by 58 ± 18%, significantly less than controls [*t*_(__4__8__)_ = 4.9, *p* < 0.0001]. The overall reduction in rate-modulation to ILD in neurons from CHL animals results in shallower rate-ILD functions. This is due to an elevation of the discharge rate at the minima of the ILD function, with no change to the maximum discharge rate. This suggests that CHL induced an overall reduction in inhibition in ICC neurons.

**FIGURE 3:**
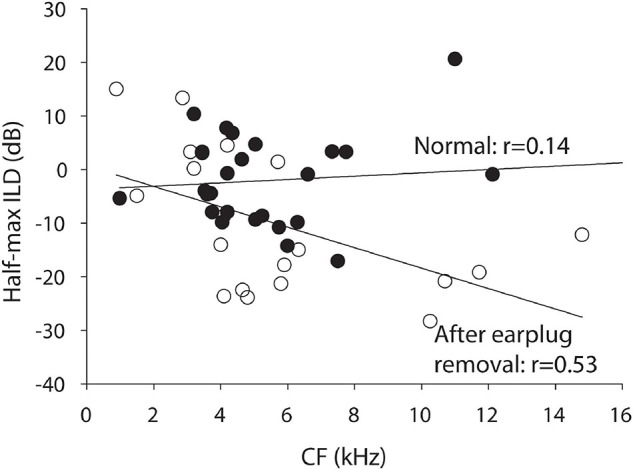
Half-maximal ILD as a function of CF for the normal control animals (black circles) and the experimental animals after 6 weeks of unilateral CHL (open circles).

Finally, linear regression of the ILD tuning curve properties considered above against the CF of the neuron were conducted. The earplugs produced larger CHL at frequencies ∼ > 4 kHz than those below and it is possible that binaural neurons with higher CF might have been more impacted by the earplug than neurons with lower CF. Of all of the regression analyses, only one test was significant. Figure 3 shows that the half-max ILD was shifted significantly more for neurons of higher CF than lower after earplug removal (*r* = 0.53, *p* = 0.02); there was no correlation (*r* = 0.14, *p* = 0.47) between half-max ILD and CF in the control animal neurons, consistent with what we have reported before in a much more extensive dataset (Jones et al., 2015).

### Inferior Colliculus Responses Carry Less Information Regarding Interaural-Level Difference Cues Following Conductive Hearing Loss

**FIGURE 4:**
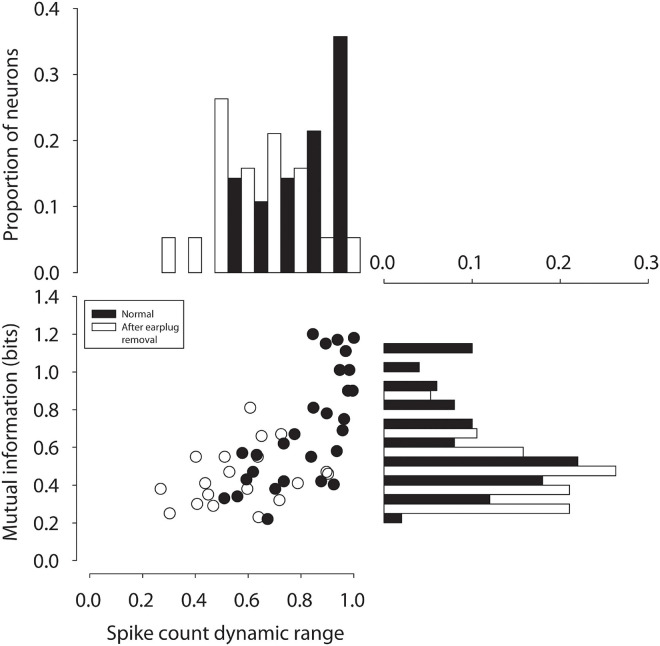
Mutual information between spike count and ILD in ICC neurons in normal-hearing (black circles) adults and after 6 weeks of unilateral CHL (open circles) is plotted as a function of the spike count modulation dynamic range by ILD (% re: max count).

Figure 4 shows mutual information computed for 31 neurons from control animals (black bars, symbols) and for 19 neurons measured from CHL animals in the ICC contralateral to the CHL (white bars and symbols). Neurons from control animals exhibited a mean MI of 0.7 ± 0.29 (median: 0.64) bits, whereas neurons from experimental animals (mean: 0.44 ± 0.15; median: 0.41 bits) were significantly lower [*t*_(__4__8__)_ = −3.4, *p = * 0.0015]. The responses of ICC neurons thus carried ∼37% less mutual information regarding ILD cues following a unilateral CHL as compared to normal-hearing controls. For neurons contralateral to the CHL, reduction in the information-carrying capacity was consistent with the significant reduction in the amount by which ILD modulated the spike count relative to the max count revealed in the earlier section. This is consistent with an effective reduction in inhibition due to the CHL. While there was a significant correlation between the half-max ILD and CF of neurons after earplug removal, there was no significant correlations between either MI or spike count modulation and neuron CF.

## Discussion

Altered inputs to the auditory system can result in anatomical, physiological, and behavioral changes that persist long beyond resolution of the hearing impairment (reviewed by Moore and King, 2004; Tollin, 2010; Whitton and Polley, 2011; Kumpik and King, 2019). The majority of evidence for CHL-induced plasticity in the auditory system comes from developmental studies in humans and animals. However, studies in adult humans and animals have also suggested that CHL can induce plasticity and that subjects can adapt to altered auditory inputs particularly via behavioral training paradigms. The data presented here suggests a compensatory mechanism for plasticity by at least the level of the inferior colliculus (ICC) as well as reduced information processing of ICC neurons. Figure 2 illustrates our general hypothesis regarding compensatory changes in the ascending circuitry to the IC in response to a unilateral CHL. In normal-hearing circuitry (Figure 2A, solid lines), spike rate is modulated by ILD sigmoidally with maximum responses for ILDs favoring the excitatory contralateral ear and reduced responses for ILDs favoring the inhibitory ipsilateral ear. Immediately after introduction of a CHL (in this case, earplug insertion), the rate-ILD curves would shift toward the right simply because the acoustical input from the contralateral ear has been attenuated (i.e., less effective excitatory input). This is represented by the dashed lines in Figure 2A.

Figure 2B illustrates the hypothesized circuit changes that would occur if mechanisms were to compensate for the altered sound localization cues due to CHL (see Lupo et al., 2011; Thornton et al., 2012, 2013). Compensatory mechanisms could potentially work to shift the rate-ILD curves back toward normal (dashed line overlapping normal curves). To achieve this kind of adaptive compensation in the ICC contralateral to the CHL, the strength (or gain) of inhibitory input from the ipsilateral normal-hearing ear (left side in example) is hypothesized to be reduced and/or the strength (or gain) of the excitation from the contralateral CHL-ear increased; the size of the “+” and “−” symbols have been adjusted in Figure 2B to illustrate this change. After removing the CHL, the effective changes to the ILD-coding pathways to the ICC can be revealed. If the circuit had been altered as in Figure 2C, then after CHL removal the rate-ILD curves are hypothesized to shift toward the left (Figure 2C, black dashed line, left column), demonstrating a reduced ipsilateral inhibitory (and/or increased contralateral excitatory) response when compared to normal. Our data is consistent with this hypothesis. Figure 3 shows that after earplug removal the shift was larger for neurons with higher CF than lower. These results are consistent with the hypothesis that the earplugs induced larger CHL for higher frequencies than lower and that the compensation for the CHL by the circuit was larger at higher CFs.

A similar compensatory response is expected in ICC neurons that are ipsilateral to the CHL (Figure 2, right panels), although insufficient data were collected in these studies to test this hypothesis. Immediately after introduction of a CHL, there will be an effective reduction in the strength of inhibition to the ICC ipsilateral to the CHL simply due to the attenuation of sound (Figure 2A, dashed line, right column). If adaptive compensation occurs, the strength of excitatory contralateral inputs will be reduced in order to match the reduced inhibitory inputs and/or an increase in the strength of the ipsilateral inhibitory input to match the normal contralateral excitation. These changes would effectively shift the rate-ILD curves back to normal with the CHL in place (Figure 2B, dashed line, right column). Immediately after CHL removal, an overall large inhibitory response would remain, causing the rate-ILD curves to shift to the left of normal (Figure 2C, dashed line, right column). Future studies will be required to test these predictions.

The present results are inconsistent with some previous physiological results in the ICC of animals with unilateral CHL (summarized by Moore and King, 2004; Tollin, 2010; Whitton and Polley, 2011). Prior studies in rats demonstrated that CHL persistently reduced the effectiveness of inputs to the two ICCs from the ear with the CHL, a finding that produces data consistent with illustrations in Figure 2A as opposed to Figure 2C (Clopton and Silverman, 1977; Silverman and Clopton, 1977; Popescu and Polley, 2010). One possible reason for this may be that the experiments in the current study were performed in the chinchilla which is a precocious species (Jones et al., 2011a, b) that also has good low-frequency hearing. Additionally, the results of the prior studies could potentially be due to an altered periphery due to the CHL, where there might be a residual hearing loss due to peripheral damage due to the CHL even after CHL removal, which would also yield results as in Figure 2A (dashed lines) even without central auditory-system plasticity. However, the most plausible explanation for the difference is that the prior studies were conducted in developing animals that were earplugged at or around hearing onset, while the present studies were done in audiologically mature young adult animals. More studies are needed to reveal the sources of the differences in the results.

While compensatory plasticity may or may not occur as illustrated in Figure 2, there is no doubt that CHL exerts an effect on the neural coding of spatial information in ICC neurons that persists after CHL resolution, as demonstrated by the 37% reduction in the capacity of neurons to carry information about ILDs (Figure 4). Reduced MI may suggest alterations in the responsiveness (spike rates), reliability (spike rate variability), as well as the general sensitivity of ICC neurons and/or their inputs to the cues to location, including ILD. The results suggest that at least for ICC neurons contralateral to the CHL, a reduction in the capacity of ILD to modulate spiking was correlated with a reduction in information-carrying capacity of these neurons. The impaired neural information processing demonstrated here may provide a basis for the persistent behavioral deficits in binaural and spatial hearing tasks that have been observed clinically after chronic CHL both during development and in adulthood. Since we have found persistent reductions in the capability of critical neural circuits in the ascending auditory pathway to encode spatial attributes of sound, it may logically follow that there will be a similar reduction in the perceptual capabilities as well. One caveat of this study is that the effects of the unilateral CHL were studied within 24–36 h after earplug removal. It is possible that the effects we observed were transient, and the binaural deficits may resolve over time. Toward this end, ongoing studies are examining the behavioral consequences of reduced information processing due to CHL induced during both development and into adulthood as well as longitudinal studies post CHL resolution.

## Data Availability Statement

The raw data supporting the conclusions of this article will be made available by the authors, without undue reservation.

## Ethics Statement

The animal study was reviewed and approved by the University of Colorado Anschutz Medical Campus Animal Care and Use Committees.

## Author Contributions

JT and DT designed the research and secured the funding. JT, KA, and DT collected, analyzed the data, and wrote the manuscript. All authors contributed to the article and approved the submitted version.

## Conflict of Interest

The authors declare that the research was conducted in the absence of any commercial or financial relationships that could be construed as a potential conflict of interest.

## Publisher’s Note

All claims expressed in this article are solely those of the authors and do not necessarily represent those of their affiliated organizations, or those of the publisher, the editors and the reviewers. Any product that may be evaluated in this article, or claim that may be made by its manufacturer, is not guaranteed or endorsed by the publisher.
